# Adjusting HIV Prevalence for Survey Non-Response Using Mortality Rates: An Application of the Method Using Surveillance Data from Rural South Africa

**DOI:** 10.1371/journal.pone.0012370

**Published:** 2010-08-25

**Authors:** Makandwe Nyirenda, Basia Zaba, Till Bärnighausen, Victoria Hosegood, Marie-Louise Newell

**Affiliations:** 1 Africa Centre for Health and Population Studies, University of KwaZulu-Natal, KwaZulu-Natal, South Africa; 2 Department of Population and International Health, Harvard School of Public Health, Boston, Massachusetts, United States of America; 3 Centre for Population Studies, London School of Hygiene and Tropical Medicine, London, United Kingdom; 4 Centre for Paediatric Epidemiology and Biostatistics, UCL Institute of Child Health, London, United Kingdom; University of Cape Town, South Africa

## Abstract

**Background:**

The main source of HIV prevalence estimates are household and population-based surveys; however, high refusal rates may hinder the interpretation of such estimates. The study objective was to evaluate whether population HIV prevalence estimates can be adjusted for survey non-response using mortality rates.

**Methodology/Principal Findings:**

Data come from the longitudinal Africa Centre Demographic Information System (ACDIS), in rural South Africa. Mortality rates for persons tested and not tested in the 2005 HIV surveillance were available from routine household surveillance. Assuming HIV status among individuals contacted but who refused to test (non-response) is missing at random and mortality among non-testers can be related to mortality of those tested a mathematical model was developed. Non-parametric bootstrapping was used to estimate the 95% confidence intervals around the estimates. Mortality rates were higher among untested (16.9 per thousand person-years) than tested population (11.6 per thousand person-years), suggesting higher HIV prevalence in the former. Adjusted HIV prevalence for females (15–49 years) was 31.6% (95% CI 26.1–37.1) compared to observed 25.2% (95% CI 24.0–26.4). For males (15–49 years) adjusted HIV prevalence was 19.8% (95% CI 14.8–24.8), compared to observed 13.2% (95% CI 12.1–14.3). For both sexes (15–49 years) combined, adjusted prevalence was 27.5% (95% CI 23.6–31.3), and observed prevalence was 19.7% (95% CI 19.6–21.3). Overall, observed prevalence underestimates the adjusted prevalence by around 7 percentage points (37% relative difference).

**Conclusions/Significance:**

We developed a simple approach to adjust HIV prevalence estimates for survey non-response. The approach has three features that make it easy to implement and effective in adjusting for selection bias than other approaches. Further research is needed to assess this approach in populations with widely available HIV treatment (ART).

## Introduction

About 33 million people were estimated to be HIV infected worldwide in 2007 [1]. Despite the relative ease of diagnosing HIV in adults, even in developed countries, the exact number of HIV infected people is unknown because not all those contacted in population surveys or surveillance systems will consent to HIV testing [2]. Traditionally population estimates of HIV prevalence in sub-Saharan Africa have been based on sentinel surveillance of pregnant women attending antenatal clinics [3], [4]. These data remain widely available and used particularly in resource poor settings, even though they are known to be biased due to lower fertility of HIV positive women, and in some countries, by unrepresentative selection of surveillance clinics [5].

Many other sources have been utilised in more recent times such as regional or national household surveys [6], surveys among high-risk populations [7], [8], and population-based surveillance studies [9]. A common feature of many of these surveys is non-response, and a major concern for analysis and generalisation is that the level of non-response can result in substantial biases in the population HIV prevalence estimates [10]. This is of particular concern if differential response rates are associated with specific characteristics of the population or high-risk groups [11] and if these data are used as inputs for deriving demographic, social and economic impacts of HIV.

We previously examined mortality patterns and levels by HIV infection status in rural South Africa using data from three annual population-based HIV surveys conducted between 2003 and 2006 [12]. The age-adjusted mortality rate in 2005 among HIV-infected adults (15–54 years) was reported at 53.9 deaths per 1,000 person-years and among HIV uninfected adults as 4.6 deaths per 1,000 person-years; the age-adjusted mortality among adults contacted but who refused to test in the HIV surveillance was estimated to be 26.2 deaths per 1,000 person-years. In the analysis here we use results of the HIV surveillance conducted by the Africa Centre in 2005 in the Umkhanyakude area of KwaZulu-Natal in which 58% eligible people refused to participate in the surveillance, to suggest a method for estimating the effect of gender-specific refusal rates on HIV prevalence estimates.

High levels of test refusal are not unique to our research area, and are common in HIV surveillance in South Africa, as highlighted by García-Calleja et. al. [10]. A review of 20 population-based HIV prevalence surveys conducted between 2001 and 2005 in 19 sub-Saharan countries including South Africa showed that the proportion of women who refused HIV testing in the surveys ranged from 1% to 17% in 18 of the 19 countries but in South Africa the refusal rate was 30% (women) and 35% (men) [10]. Further, within South Africa, a recent nationally representative HIV survey found KwaZulu-Natal province (where our study area is located) to have the highest refusal to HIV testing at 37% (excluding absentees and non-contacts) [13].

The aim of this paper is to describe a simple model which uses mortality rates by HIV status to estimate HIV prevalence among the population who refused to participate in the HIV surveillance. The estimated prevalence among those who refused testing is then used to adjust the overall prevalence in the total population in the area. We use longitudinal demographic surveillance data from rural South Africa to derive mortality rates for persons who tested negative and positive during HIV surveillance in the same area in 2005 (testers), and for those who refused to participate in the 2005 HIV surveillance (non-testers) and for whom HIV status was thus unknown to us. The model uses these mortality rates, and assumes that mortality among the HIV status unknown group is a weighted average of the mortality rates of those whose HIV status was known, to estimate HIV prevalence among non-testers. Given that the data are from a longitudinal demographic surveillance with routine recording of deaths and the population at risk, the resulting mortality data could provide a tool for adjusting HIV prevalence in the study population to allow for refusal to test. This method could be applicable in other demographic surveillance sites, with relatively high rates of refusal to participate in HIV testing but reliable mortality data for the population which can be categorised by HIV status.

We compare observed HIV prevalence rates (from those who consented to testing) to the adjusted HIV prevalence rates by age and sex, and document a significant underestimate in the observed prevalence rates.

## Methods

### Data sources

Data used in this analysis, and the initial work that motivated it, come from the longitudinal Africa Centre Demographic Information System (ACDIS), located in a largely rural district of Umkhanyakude in northern KwaZulu-Natal, South Africa (www.africacentre.com). The ACDIS has two components that run parallel, the bi-annual household surveillance and the annual individual surveillance. Demographic, social and economic data have been collected since 2000 from key informants reporting on all individual household members whether resident or non-resident in the geographically well-defined surveillance area in the household surveillance. In the individual surveillance, which has been conducted since 2003, information on health and sexual behaviours and a blood sample for HIV sero-status testing is collected [14], [15] from women 15–49 and men 15–54 years and a dried blood spot is prepared from a finger prick. A broad based HIV-1/HIV-2 ELISA test (Vironostika, Organon Teknika, Boxtel, The Netherlands) is used to determine HIV status at the Centre's virology laboratory in Durban. All positive test results are confirmed by a second ELISA (GAC-ELISA, Abbott, Abbott Park, Illinois, USA) on the same sample. HIV infection is defined by positive antibody status on both ELISAs, ‘HIV negative’ status was defined by a negative first ELISA. Those not consenting to HIV testing were classified as ‘HIV unknown’.

### Ethics statement

During household visits with eligible individuals in the individual surveillance, written informed consent is obtained for the collection, storage and use in research of the blood sample and sexual behaviour data. For household surveillance in which demographic data are collected, oral informed consent is obtained from the key informant who is usually the head of household, but in his or her absence a competent adult household member. Field workers are thoroughly trained every year and between the surveillance rounds to ensure they offer both written and oral informed consent in a uniform manner. Ethical approval for the individual surveillance involving written consent and the household surveillance using oral consent was obtained from the University of KwaZulu-Natal Biomedical Research Ethics Committee.

### Study sample

On January 1^st^ 2005 there were 84,964 individuals in 11,000 households under demographic surveillance; 21,472 resident individuals (women and men 15–49 years) were contacted during the 2005 HIV surveillance. Person-years of exposure were estimated from the date at which an individual was HIV sero-tested in 2005, or from the date of visit for individuals contacted but who refused to participate in the HIV testing, and right-censored on 31st December 2007 or by death, out-migration, or household membership ending. Mortality rates were calculated by dividing the number of deaths by the person-years of exposure for three groups of people 1) HIV-negative, 2) HIV-positive and 3) HIV status unknown (i.e. eligible resident individual was contacted but not tested in the HIV surveillance).

We used non-parametric bootstrapping [16] with replacement over 1,000 repetitions to estimate the 95% confidence intervals associated with the HIV prevalence point estimates.

### Presentation of a simple analytical model

We used reported mortality rates by HIV status (positive, negative, unknown) to infer the HIV prevalence among those who refused to participate in the HIV surveillance. We assumed that mortality in the untested group is associated with the same factors as in the tested group, and that apart from sex, age and HIV status, mortality determinants were distributed in the untested group in the same way as in the general population. The total person-years lived in the group with unknown HIV status is contributed partly by HIV infected persons and partly by uninfected persons. Using this understanding and the assumptions above, the simple analytical model can be presented as follows.

Allowing for mortality in the HIV negative, HIV positive and HIV unknown group to vary by sex, s, age group, a, and time period, t, and denoting mortality rates by M, and using the subscripts p, n and u to denote the rates in persons with positive, negative and unknown HIV status respectively:
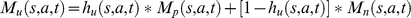
(1)where ***h_u_(s,a,t)*** is the proportion of total person-years lived in the HIV unknown population (of sex s, age a, time t) contributed by infected persons. Another name for h_u_ is the period HIV prevalence in the untested population. ***h_u_(s,a,t)*** is unknown, but all the mortality rates are known as ***M_p_***, ***M_n_*** and ***M_u_*** have been measured in the demographic surveillance system. Thus, we can solve for ***h_u_(s,a,t)***,:
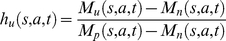
(2)We can also compare the period HIV prevalence in the untested population ***h_u_(s,a,t)***, with period HIV prevalence in the population whose status is known, ***h_k_(s,a,t)***, because the latter is the person-years lived by those who tested positive as a fraction of the person-years lived by positive and negative people.
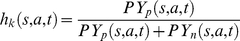
(3)An adjusted estimate, ***h_w_(s,a,t)***, of the HIV prevalence in the whole population can be obtained as a weighted sum of the estimated period HIV prevalence in the untested population and the observed HIV prevalence among those who tested, weighting by the person-years lived by the tested and the untested populations.
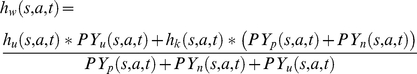
(4)


## Results

**Figure 1:**
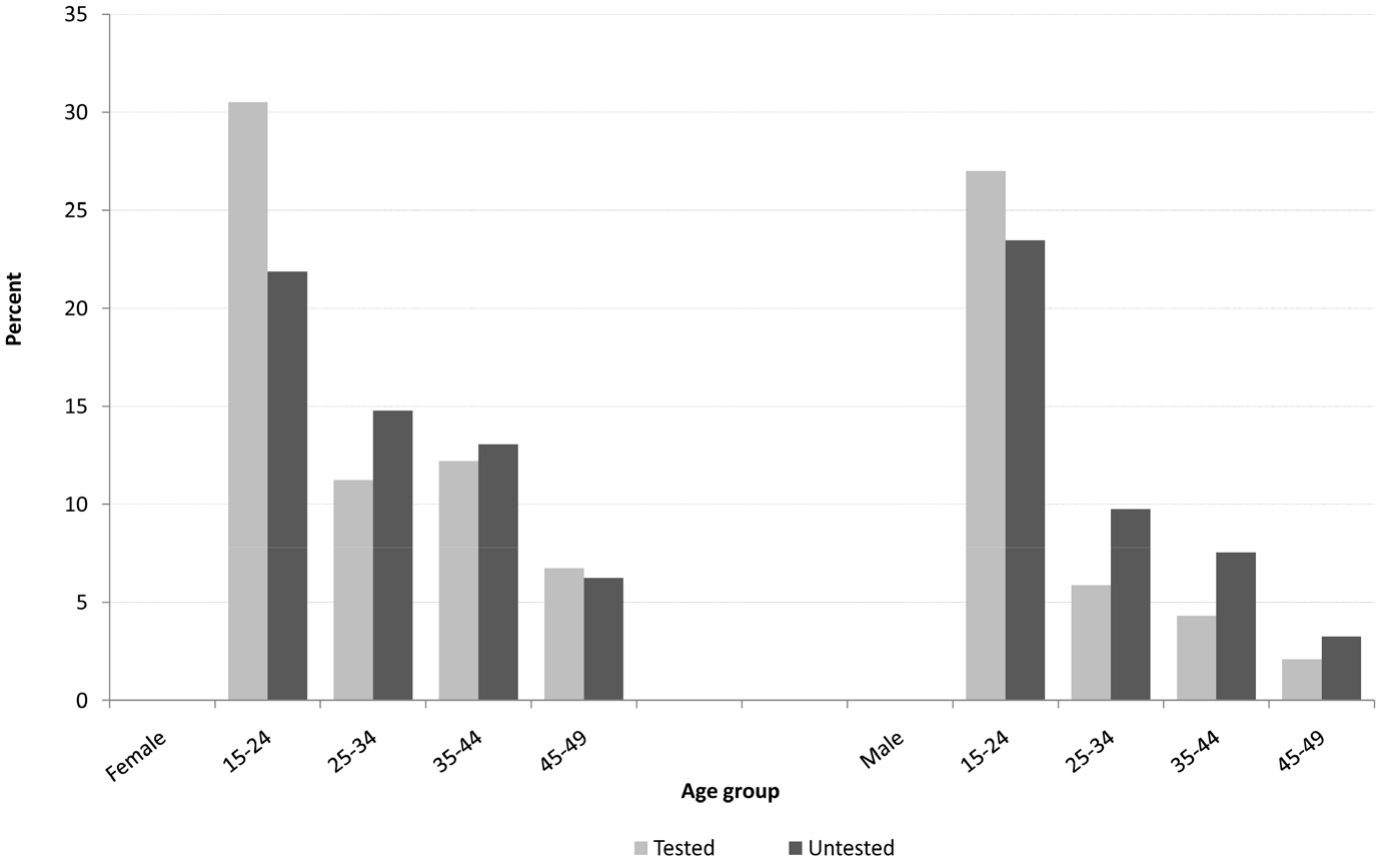
Distribution of tested and untested by age and sex, ACDIS 2005 HIV survey.

Overall, 59% of the 21,305 resident individuals did not provide a sample for HIV testing in the 2005 round, and their HIV status was marked as unknown; slightly more females 54% (n = 6846) than males 46% (n = 5762) were in this unknown HIV status category. Table 1 gives the consent rates by sex among eligible adult residents in the ACDIS annual HIV surveys between 2003 and 2006. The proportion of men refusing to participate in the HIV survey is higher than that of females in each year. By age refusal to participate was relatively high in the age range 25–44 years, which is also a peak prevalence age range, which in itself is likely to bias downwards the crude observed prevalence derived from those tested. Figure 1 shows that those who did not test, for males and for females, had an older age distribution than those who tested, except for the 15–24 age group. The adjustment method proposed here goes further than identifying differences in age and sex composition of those who agree to participate and those who refuse, looking within specific age- and sex- groups for evidence of systematic differences between the tested and those who refused.

**Table 1:** Participation rates among residents aged 15–54years in HIV testing, 2003–6.

	Female		Male		Both sexes	
Survey year	n	(%)	n	(%)	n	(%)
2003/4	12,259	59	9,010	56	21,269	58
2005	12,011	43	9,294	38	21,305	41
2006	12,537	40	9,452	34	21,989	38

**Figure 2:**
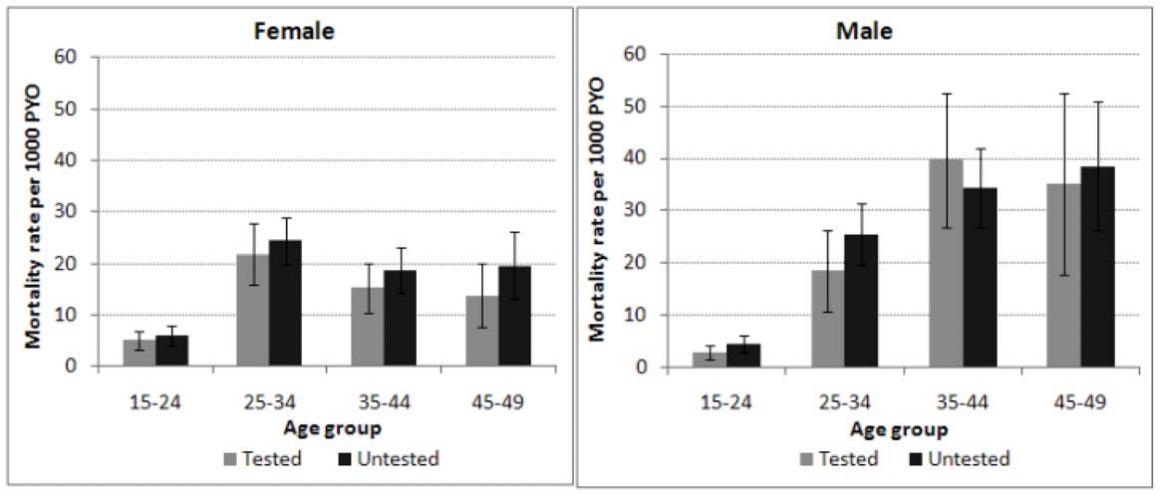
Mortality patterns by age and sex between tested and untested participants, 2005.

A comparison of mortality rates by age and sex of the population who tested versus the untested (Figure 2), suggests that those who tested had higher mortality than those who did not. Mortality rates were higher among the untested (16.9 per thousand person-years) than the tested population (11.6 per thousand person-years). By individual age groups and sex, females who did not test had higher mortality than those who tested, with the difference in the age group 45–49 being statistically significant (Figure 2). A similar pattern was observed for males, except for the age group 35–44 years, where those who did not test had a lower mortality than those who tested. For both sexes combined, the differences were marginally significant in the age groups 25–34 and 45–49 years respectively.

The individual age group differences do not reach statistical significance, but overall mortality is significantly higher in the untested group than in the tested, due not only to differences in the age and sex composition of the tested and untested groups, as can be shown by direct standardisation. Applying the age- and sex- specific mortality rates in the tested group to the age-sex composition of the total population we obtain a standardised mortality rate of 13.7 per thousand (12.6, 14.7) whereas the standardised rate resulting from applying the age- and sex- specific rates of the untested population is 16.2 per thousand (15.1, 17.3). Since the overall difference between the crude mortality rates in the tested and untested populations is 4.8 per thousand ( = 15.9 - 11.1), as shown in Table 2, and the standardised difference is 2.5 per thousand ( = 16.2 - 13.7), we can attribute a difference of 2.3 per thousand to the age- and sex composition difference between the tested and untested populations, and a difference of 2.5 per thousand to actual mortality differences between the tested and untested groups. Assuming that this mortality difference can be explained by differences in HIV infection rates between the tested and untested groups we investigate the implied HIV prevalence in the untested group, and hence deduce an adjusted value for overall prevalence, allowing for the effects of test refusal. We make these estimates separately for females and males and by age group, with 95% confidence intervals estimated using a bootstrap resampling method.

**Table 2:** Person-years, mortality rates and HIV prevalence rates by age and sex, 2005.

	*Negative*				*Positive*				*UnTested*				*Tested*			
Female	PYO	MR	[95%	CI]	PYO	MR	[95%	CI]	PYO	MR	[95%	CI]	PYO	MR	[95%	CI]
15–24	5,325	2.4	1.1	3.8	888	21.4	11.8	31.0	6,260	5.9	4.0	7.8	6,214	5.2	3.4	6.9
25–34	1,211	3.3	0.1	6.5	1,078	42.7	30.3	55.0	4,229	24.4	19.7	29.0	2,289	21.8	15.8	27.9
35–44	1,710	2.9	0.4	5.5	774	42.6	28.0	57.2	3,745	18.7	14.3	23.1	2,484	15.3	10.4	20.2
45–49	1,090	5.5	1.1	9.9	286	45.4	20.5	70.4	1,787	19.6	13.1	26.1	1,376	13.8	7.6	20.0
Total	9,336	3.0	1.9	4.1	3,027	36.7	29.8	43.5	16,021	15.3	13.4	17.2	12,363	11.2	9.4	13.1
**Male**																
15–24	5,333	2.3	1.0	3.5	166	18.1	−2.5	38.6	6,719	4.5	2.9	6.1	5,499	2.7	1.3	4.1
25–34	779	6.4	0.8	12.1	416	40.8	21.4	60.3	2,793	25.4	19.5	31.3	1,195	18.4	10.7	26.1
35–44	582	12.0	3.1	21.0	299	93.6	58.7	128.5	2,162	34.2	26.4	42.0	881	39.7	26.5	52.9
45–49	323	15.5	1.8	29.1	105	94.8	35.5	154.2	935	38.5	26.0	51.0	429	35.0	17.2	52.8
50–54	319	25.1	7.8	42.4	71	112.6	34.1	191.2	742	51.2	35.2	67.1	390	41.0	21.0	61.0
Total	7,337	5.0	3.4	6.7	1,058	62.4	47.3	77.4	13,352	18.6	16.3	21.0	8,394	12.3	9.9	14.6
**Both sexes**																
15–24	10,658	2.3	1.4	3.3	1,054	20.9	12.2	29.6	12,979	5.2	3.9	6.4	11,713	4.0	2.9	5.2
25–34	1,990	4.5	1.6	7.5	1,494	42.2	31.7	52.6	7,022	24.8	21.1	28.5	3,485	20.7	15.9	25.4
35–44	2,292	5.2	2.3	8.2	1,073	56.8	42.5	71.2	5,907	24.4	20.4	28.4	3,366	21.7	16.7	26.7
45–49	1,413	7.8	3.2	12.4	392	58.7	34.5	82.9	2,722	26.1	20.0	32.1	1,805	18.8	12.5	25.2
Total	16,354	3.5	2.6	4.4	4,014	42.1	35.8	48.5	28,631	15.9	14.5	17.4	20,368	11.1	9.6	12.5

*PYO = Person-years of observation; M = mortality rate per 1000 person-years of observation*.

### Prevalence estimates by age and sex, 2005

**Figure 3:**
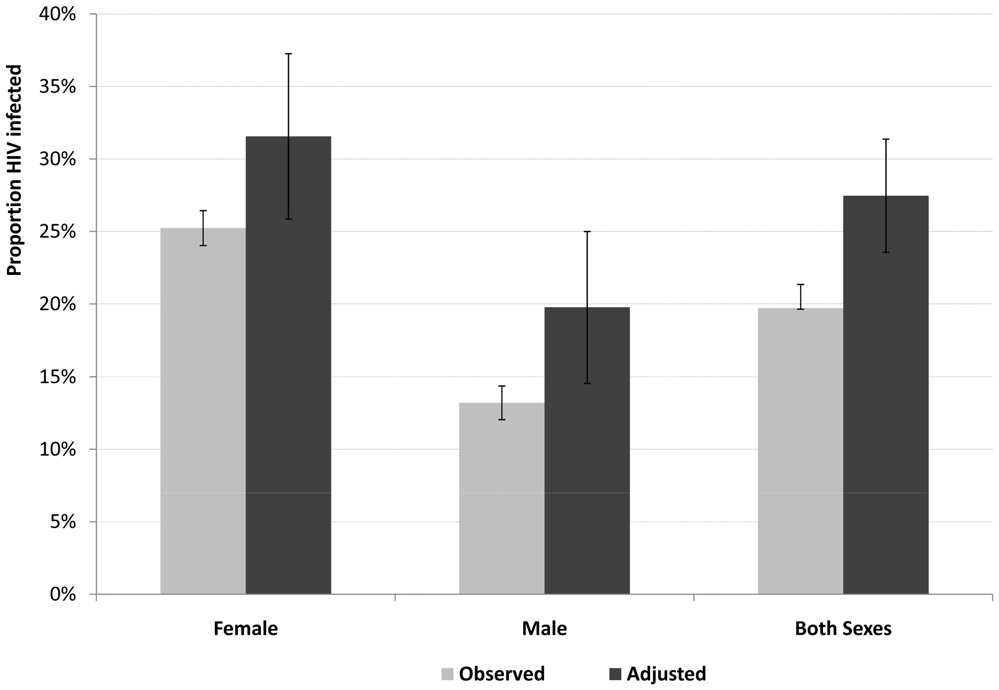
Observed and Adjusted HIV prevalence rate in adults 15–49 years by sex, 2005.

**Figure 4:**
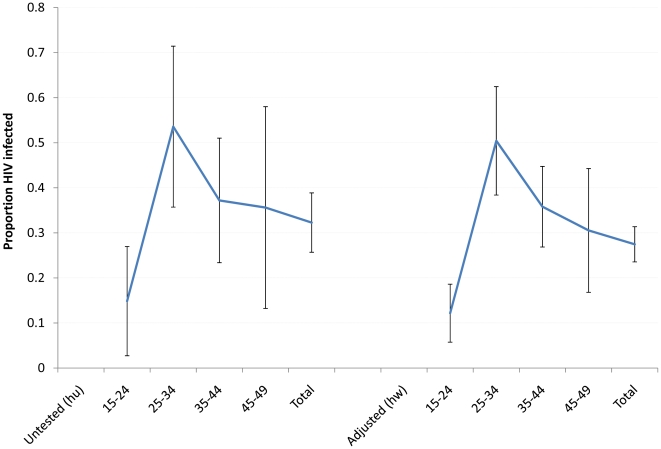
HIV prevalence rate in adults 15–49 years untested and overall adjusted, 2005.

Using equation 4 above, results shown in Figure 3, the adjusted HIV prevalence for females aged 15–49 years in 2005 was 31.6% (95% CI 26.1–37.1) compared to an observed prevalence of 25.2% (95% CI 24.0–26.4). For males aged 15–49 years the adjusted and observed prevalence was 19.8% (95% CI 14.8–24.8) and 13.2% (95% CI 12.1–14.3) respectively. For both sexes combined, the adjusted HIV prevalence was 27.5% (95% CI 23.6–31.3), compared to an observed prevalence among resident adults 15–49 years who participated in the 2005 survey of 19.7% (95% CI 19.6–21.3). Figure 3 strongly suggests that the model presented here results in a significant upwards adjustment of the observed prevalence for males and for both sexes combined as the 95% confidence limits for the adjusted prevalence estimate are outside the confidence limits of the observed prevalence from those tested. The adjusted HIV prevalence patterns by age for males and females were broadly similar to the age pattern of the overall adjusted prevalence for both sexes combined, as shown in Figure 4. Prevalence was highest in the 25–34 years age range (Table 3).

**Table 3:** Observed and Adjusted HIV prevalence rate in adults 15–49 years, 2005.

	Observed HIV pre-valence			Adjusted prevalence among untested			Adjusted overall HIV prevalence		
Age group	Prevalence (%)	95% CI		Prevalence (%)	95% CI		Prevalence (%)	95% CI	
15–24	9.0%	8.3	10.0	14.9	3.0	26.7	12.2	5.9	18.5
25–34	42.9%	41.5	46.4	53.6	34.9	72.2	50.4	37.8	63.0
35–44	31.9%	30.9	35.7	37.2	24.0	50.4	35.8	27.3	44.3
45–49	21.7%	19.6	24.7	39.8	16.9	62.8	33.0	18.9	47.0
Total	19.7%	19.6	21.3	32.3	25.7	38.8	27.5	23.6	31.3

Given the mortality rates observed by HIV sero-status, and in those of unknown HIV status in particular, the adjusted population HIV prevalence level after allowing for the likely prevalence level among the non-testers suggests an under-estimation in the observed HIV prevalence rate of around 7 percentage points for females, males and both sexes combined or a relative difference of about 28% for females (32 vs 25), 54% for males (20 vs 13) and 37% (27.5 vs 20) for both sexes. The relative difference in the prevalence rates for males should however be interpreted with caution as they were sensitive to the small numbers of males participating in HIV testing in the surveillance on one hand and their relatively high mortality on the other.

## Discussion

In this analysis we set out to explore a simple model to obtain an adjusted estimate of HIV prevalence in a population with a high rate of non-response, using HIV surveillance data and where mortality data are available and reliable. A major benefit of the model presented here is that it does not rely on complex computer simulations, or very detailed demographic data. It is simple to apply in a DSS setting as the only data required are on HIV testing status and mortality rates separately estimated by HIV status. Other more complex procedures are available to address non-response problems such as (multiple) imputations [17] and regression equations [9]. However, these methods tend to rely on individual-level variables such as mobility, marital status, work status, alcohol use, number of partners, age at first sex, concurrency and religiosity [6], which are subject to much more reporting bias than mortality data that we use in our method. These individual-level variables, unlike mortality, are in addition not routinely or uniformly collected in many surveillance sites. Furthermore, unlike the individual-level analysis that is adopted in most DHS to adjust the prevalence estimates for test refusal which require predicting the probability of an individual non-tester being infected based on his/her peculiar characteristics, the method we propose here is an aggregate model which does not suffer from the limitations of individual-level analyses. Our mortality differentials suggest that non-testers are significantly different from testers, and thus our results are different to Mishra et. al. because a) non-testers appear to have relatively higher prevalence rates than testers and b) we have a higher refusal rate than any of the studies cited by Mishra et. al.

Figure 4 shows the confidence intervals to be relatively narrow for the overall adjusted rate, but very wide in the age groups 25–34 and 45–49 years, suggesting greater uncertainty in the adjusted estimates for these age groups. This would suggest that prevalence rates in the age groups 25–34 and 45–49 years are more of an under-estimate than in other age-groups. The former age group is associated with higher HIV prevalence but relatively lower participation in HIV surveillance, while the latter age group is associated with high mortality and generally high participation rates in HIV testing. Particularly for the 45–49 years age group, the high uncertainty could be partially explained by the small numbers. The overall adjusted HIV prevalence rates for females and males as well as by age from application of our proposed model were consistently above the upper confidence limits of the observed prevalence rates. We show from this approach that the prevalence in those who consented to HIV testing in the 2005 survey under estimated the prevalence in the population as a whole by around seven percentage points. Our results suggesting the true HIV prevalence in the study population is likely under-estimated if only the observed data are considered, are in line with other studies [6], [9].

Data from our research site shows that relative to individuals who had tested negative in the first HIV round, persons who had tested positive were 23% significantly less likely to test again in the 2005 survey round. A recent study using longitudinal data from Malawi to examine the effect of prior knowledge of being HIV infected on consent to subsequent HIV surveillance testing found that Malawians who knew their positive HIV status were five times more likely to refuse participation than those who had tested negative previously [18]. A positive association between age, urban residence, being employed and absenteeism from home with refusal to participate in HIV sero-testing survey has also been shown [6], [19]. We are therefore confident that the suggested under-estimate in the observed HIV prevalence rate is valid, and our mortality-based procedure provides a reasonable method of adjustment of the observed HIV prevalence rates.

Our approach may have utility even in the context of HIV treatment (ART), although it would require making a further assumption that the proportion of infected people on treatment amongst those testing and those refusing to test in the surveillance round are broadly similar. An alternative approach would be to divide the population into four groups rather than three: those tested HIV negative, those tested HIV positive but not on treatment, those on ART regardless of whether they consented to testing or not, and those who did not test and were not on ART. Such an approach would only be feasible if ART clinic data could be linked to demographic surveillance data. There is a future opportunity in the study setting with the increasing availability of HIV treatment [20] to validate this model post-HIV treatment roll-out.

### Limitations

A limitation of this simple model is that it is dependent on the population consenting to participate in HIV surveillance not being significantly different from those not consenting with respect to factors other than HIV that determine mortality. Non-response is a common feature of population-based HIV surveys [9], [10], [11]. If non-response is particularly high among a select group or persons with particular characteristics, that pre-dispose them to lower or higher mortality from HIV, such as disease stage [21], or causes other than HIV it is likely to bias this adjustment procedure. This is why it would be useful to apply the adjustment procedure in more homogenous social categories by breaking down by residence and education - this was not possible in the present study because of small numbers. Even data from nationally representative studies such as by the Human Sciences Research Council of South Africa (HSRC) [13] also suffer from problems of test refusal which is likely to bias their results. But since such studies tend to be cross-sectional, the mortality-based method we propose here can not be applied to estimate the extent of bias. In addition, it cannot be used to adjust cross-sectional sources of national HIV prevalence data where no prospective adult mortality data are available, for example the demographic and health surveys or the South African HSRC national HIV survey.

Another major limitation of this analysis was the use of information on 29% of deaths (and accounting for 36% of the exposure) with known HIV status to adjust the HIV prevalence in the nearly two-thirds of population with unknown HIV status. This was a result of the generally high refusal rate to participate in HIV surveillance in our study population. This may have biased our findings. We examined these mortality data in detail since the proposed method so crucially depends on them to check our assumption that the same type of reporting bias affects all ages and whether mortality rate differentials might be accounted for by differences in age and sex composition in the tested and untested populations. The chi-square test (not shown) for the differences in mortality rates between tested and untested in Table 2, does not attain significance in individual age groups (because of relatively small numbers), however, for both sexes combined, and for males and females of all ages (except for males aged 35–44) the mortality rates in the untested group are higher than in the tested group. Furthermore, the fact that the relationship goes in the same direction for every age-sex group (except one) adds support to the appropriateness of using the mortality rates to adjust HIV prevalence. We further urge caution in the interpretation of mortality rates and the adjusted figures particularly at the older age groups and for males as the small numbers may have affected the mortality estimates particularly at the older age groups. The availability of information to assess tested person years of observation (Table 2) as a proportion of all person years also assists in avoiding over-interpretation. Despite these limitations the proposed method remains methodologically sound.

In conclusion, after adjusting for the untested population, there was very little change in the pattern of age specific HIV prevalence rates for women or men, but a significant difference between the overall adjusted and observed prevalence (7 percentage points in absolute terms).

Our approach renders itself to easy application and should be relevant for demographic surveillance sites such as our own that have conducted HIV prevalence surveys and can classify ensuing deaths by HIV status. Application of this method in populations with wide availability of HIV treatment (ART) may require identifying people on ART as a separate group. Our approach to adjusting for selection effects by conditioning on observed factors has three highlighted advantages over other approaches. First, only aggregate mortality and HIV prevalence data are needed. Second, HIV prevalence is highly associated with mortality [12], and third, the approach is easily applicable even in settings with only one HIV sero-survey but longitudinal mortality follow-up of those who participated in the sero-survey and those who refused. These features make our approach easy to implement and effective in adjusting for selection bias than other approaches. Given the very high proportion of our population with unknown HIV status and limitations of our mortality data in general, there is an opportunity for validation of the proposed adjustment method in this analysis in other surveillance sites with lower refusal rates and reliable mortality data. This method appears to be methodologically sound hence such a validation exercise will help to determine its reliability and robustness.
